# Gestational diabetes induces autistic-like behaviors in offspring by disrupting the GABAergic system

**DOI:** 10.3389/fnins.2025.1538115

**Published:** 2025-02-12

**Authors:** Xuan-Qi Liu, Tian-li Huang, Shu-Yu Zhang, Yu-Tong Huang, Jia-Ying Mo, Yi-Shang Yan, Yi-Ning Cao, Yue-Ran Cai, Jian-Zhong Sheng, Hong Zhu, He Feng Huang

**Affiliations:** ^1^The Key Laboratory of Reproductive Genetics (Zhejiang University), Ministry of Education, Zhejiang University School of Medicine, Hangzhou, China; ^2^The International Peace Maternity and Child Health Hospital, School of Medicine, Shanghai Jiao Tong University, Shanghai, China; ^3^Key Laboratory of Reproductive Dysfunction Management of Zhejiang Province, Assisted Reproduction Unit, Department of Obstetrics and Gynecology, Sir Run Run Shaw Hospital, School of Medicine, Zhejiang University, Hangzhou, China; ^4^The Second Affiliated Hospital, School of Medicine, Zhejiang University, Hangzhou, China; ^5^Obstetrics and Gynecology Hospital, Institute of Reproduction and Development, Fudan University, Shanghai, China

**Keywords:** gestational diabetes (GDM), autism, neurodevelopment, GABA, behavior

## Abstract

**Background:**

Increasing evidence have shown that gestational diabetes mellitus (GDM) is associated with the risk of autism in offspring. However, the underlying mechanisms have not yet been fully elucidated.

**Methods:**

A mouse model of gestational diabetes mellitus (GDM) was established to investigate its impact on offspring. Behavioral analyses were conducted to assess social novelty and stereotypic behaviors. Neuronal excitability in the prefrontal cortex (PFC) was evaluated using c-Fos staining after social behavior stimulation. Single-cell transcriptomics and metabolomics were employed to analyze changes in the GABAergic system.

**Results:**

Behavioral analyses revealed that GDM led to impaired social novelty and increased stereotypic behaviors in male offspring. c-Fos staining showed hyperexcitability in the PFC of male offspring from the GDM group following social behavior stimulation. Single-cell transcriptomics and metabolomics identified alterations in the GABAergic system, including a decrease in GABAergic neurons and reduced GABA levels. This reduction in GABA was associated with decreased GAD2 expression due to DNA hypermethylation in the GAD2 promoter region.

**Conclusion:**

These data suggest that GDM induces autistic-like behaviors, including reduced social novelty and increased stereotypic behaviors, in offspring by affecting the GABAergic system. These findings provide new insights into how GDM may influence neurodevelopment in offspring.

## 1 Introduction

Autism spectrum disorder (ASD) is a heterogeneous neurodevelopmental disorder characterized by early-appearing impairments in social interaction and communication, along with repetitive patterns of behavior, interests, or activities (Lai et al., 2014). According to the Centers for Disease Control and Prevention’s Autism and Developmental Disabilities Monitoring Network, approximately 1 in 44 children are diagnosed with ASD, with boys being four times more likely to be affected than girls (Malwane et al., 2022; Yang et al., 2022). ASD has become a significant public health concern due to its complex causes, late diagnosis, and prevention challenges. Researchers have proposed various explanations for the mechanisms underlying the highly diverse symptoms of ASD. Notably, the PFC plays a critical role in regulating social behaviors, and its dysfunction has been implicated in ASD-related symptoms. A study published in Nature in 2011 demonstrated that reducing the excitability of interneurons in the PFC of mice could rescue the social impairment phenotypes caused by E/I imbalance (Yizhar et al., 2011). Additionally, a clinical study observed through MR spectroscopy that GABA levels were significantly reduced in the brains of children with complex stereotyped movements (Harris et al., 2016). An animal experiment utilized metabolomics to demonstrate that intrauterine hyperglycemia can reduce GABA levels in the fetal brain of mice (Perez-Ramirez et al., 2024). This suggests that an intrauterine hyperglycemic environment may disrupt the E /I balance in the offspring’s brain. However, whether this effect will persist into adulthood and result in behavioral changes remains unclear.

One of the most common metabolic disorders during pregnancy, GDM, is defined as glucose intolerance that arises or is first diagnosed during pregnancy (McIntyre et al., 2019). It is primarily characterized by elevated blood glucose levels during mid to late pregnancy. The prevalence of GDM varies widely, ranging from 1 to 31% worldwide, and continues to show an increasing trend (McIntyre et al., 2019). GDM represents a major metabolic disruption in pregnancy that not only affects maternal health but also poses risks to fetal development (Sweeting et al., 2022). Epidemiological evidence strongly suggests that prenatal exposure to GDM is associated with an increased risk of neurodevelopmental disorders, including ASD, in offspring (Liu et al., 2024). A retrospective longitudinal cohort study demonstrated that children exposed to GDM have a 42% higher risk of developing ASD, with the strongest association observed in cases where GDM was diagnosed by 26 weeks’ gestation (Xiang et al., 2015). Gestational diabetes-induced intrauterine hyperglycemia can increase the risk of ASD through various potential biological mechanisms, such as fetal hypoxia (Burstyn et al., 2011), oxidative stress in umbilical cord blood and placental tissue (Chauhan et al., 2004), chronic inflammation, and epigenetic dysregulation (Schanen, 2006).

The developmental origins of health and disease (DOHaD) hypothesis provides a framework for understanding how adverse environmental exposures during critical periods of fetal development can increase susceptibility to diseases later in life (Barker et al., 1989). The late fetal stage is a critical period for neuronal migration in the neocortex, marking a key time point for the development of brain regions and neural circuits associated with autistic-like behaviors (Marín and Rubenstein, 2003; Bystron et al., 2008). This period overlaps with the onset of GDM, but studies on the underlying mechanisms are lacking.

In this study, we utilized a mid-to-late gestational intrauterine hyperglycemia mouse model to investigate how GDM affects offspring neurodevelopment and behavior. Our results demonstrate that male offspring exposed to GDM exhibit increased stereotyped behaviors and impaired social novelty, whereas these phenotypes are less pronounced in female offspring, suggesting sex-specific vulnerabilities. Further mechanistic analyses revealed elevated excitability in the PFC of male offspring after social stimulation. Single-cell RNA sequencing and metabolic profiling identified disruptions in the GABAergic system, which mediated the autistic-like behavioral abnormalities in offspring exposed to GDM. By focusing on the interaction between GDM, neural excitability, and specific autistic-like phenotypes, this study provides new insights into how GDM contributes to neurodevelopmental disorders within the framework of the DOHaD hypothesis.

## 2 Materials and methods

### 2.1 Animals

All experimental procedures in this study were approved by the Animal Ethics Committee of Zhejiang University. Adult female and male C57BL/6J mice (56 days old, weighing 30–35 g) were purchased from Shanghai SLAC Laboratory Animal Co., Ltd. (Shanghai, China). Before mating, both female and male mice were isolated for 1 week to acclimate to the experimental environment. The animals were housed in individually ventilated cages under a 12/12 h light-dark cycle, with free access to food and water in a temperature and humidity controlled facility (23–26°C, 50–60%).

**FIGURE 1:**
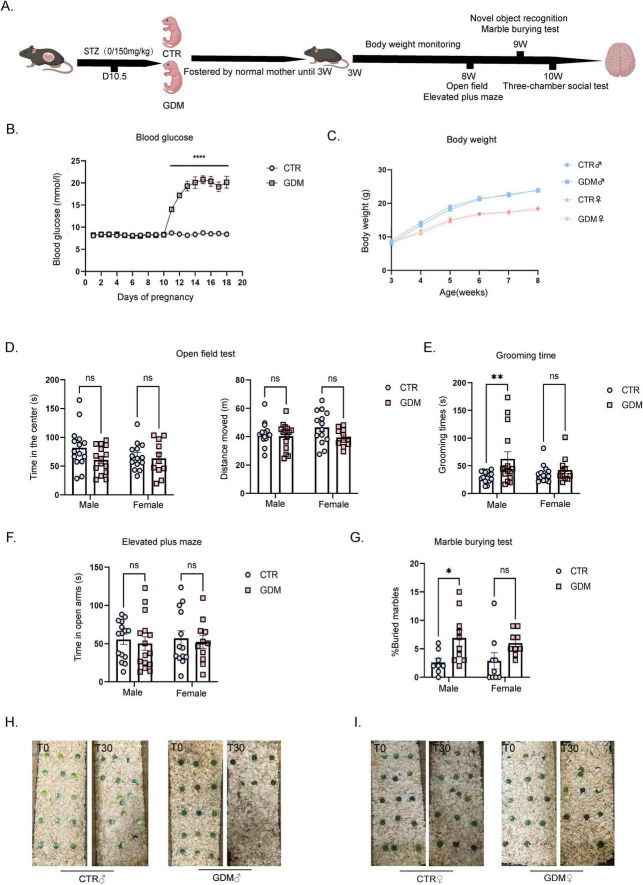
Gestational diabetes induced repetitive behaviors in male offspring. **(A)** Model diagram. **(B)** Random blood glucose during pregnancy. *n* = 6/group, F (17, 180) = 58.43, *p* < 0.0001. **(C)** Male and Female offspring body weight from 3 to 8 weeks (*n* = 6/group). **(D)** Open field test of male offspring (*n* = 15/group) and female offspring (CTR: *n* = 14; GDM: *n* = 11). **(E)** The total grooming time in the open field of male offspring (*n* = 15/group) and female offspring (CTR: *n* = 14; GDM: *n* = 11). F_interaction_ (1, 51) = 2.561, *p* = 0.1157; F_sex_ (1, 51) = 0.7493, *p* = 0.3908; F_GDM_ (1, 51) = 5.697, *p* = 0.0207, followed by Šídák’s multiple comparisons to analyze the effects produced by gestational diabetes mellitus (GDM), t = 2.96, df = 51.00, *p* = 0.0091. **(F)** The elevated plus maze test of male offspring (*n* = 15/group) and female offspring (CTR: *n* = 14; GDM: *n* = 11). **(G)** The graph shows the percentage of buried marbles of male offspring (CTR: *n* = 8; GDM: *n* = 10) and female offspring (*n* = 9/group). F _interaction_(1, 32) = 0.2500, *p* = 0.6205; F_sex_ (1, 32) = 0.07469, *p* = 0.7864; F (1, 32) = 10.07,*p* = 0.0033, followed by Šídák’s multiple comparisons to analyze the effects produced by GDM, t = 2.589, df = 32.00, *p* = 0.0285. **(H)** Pictures of the marbles at time 0’ and 30’ of the male experimental groups are illustrated. **(I)** Pictures of the marbles at time 0’ and 30’ of the female experimental groups are illustrated. In panel **(B–G)**, data were analyzed by two-way ANOVA, followed by Šídák’s multiple comparisons. *p < 0.05, **P < 0.01, ****p < 0.0001. Data are presented as mean ± SEM.

The GDM modeling method was modified from our team’s previous protocol (Zhu et al., 2019). Since the original model used ICR mice, our experiment employed C57/BL6J mice to achieve results more closely aligned with human conditions (Waterston et al., 2002). C57BL/6 mice are a commonly used inbred strain, but they exhibit relatively low reproductive performance (Ayadi et al., 2011). For C57/BL6J mice, the modified single-dose intraperitoneal injection of STZ, compared to the two-dose method used in the original model (administered on gestational days 6.5 and 11.5), results in a higher litter rate while achieving the same standard increase in gestational blood glucose levels. The day of vaginal plug detection was defined as gestation day (GD) 0.5. Pregnant female mice were randomly divided into two groups: control group (CTR) and GDM group. On gestation day 9.5, the mice were fasted overnight (8–12 h), and on gestation day 10.5, the pregnant mice in the GDM group were given an intraperitoneal injection of streptozotocin (STZ; 150 mg/kg; Sigma-Aldrich, St. Louis, MO, United States, S0130), while the CTR group received an equivalent dose of citrate buffer (Figure 1A). Streptozotocin is a drug that is highly toxic to pancreatic β-cells, leading to the destruction of insulin-producing cells and inducing elevated blood glucose levels (Rossini et al., 1977). It is widely used to simulate gestational diabetes in mouse models. Blood glucose levels were measured daily starting on GD 11.5, and a blood glucose level above 14.9 mmol/L was considered indicative of successful model establishment (Figure 1B). After birth, both the offspring of the GDM and the CTR group were fostered by naturally pregnant mothers who had not undergone any treatment (neither STZ injection nor citrate buffer injection) until 3 weeks of age.

### 2.2 Behavior analysis

All behavioral tests were conducted during the light phase between 09:00 and 18:00. Behavioral testing began when the mice were 8 weeks old and concluded at 10 weeks of age, with a minimum interval of 1 day between different tests. All tests were recorded using a camera positioned above the testing arena, and the movement of each mouse was tracked using ANY-maze software. After each mouse completed testing, feces and urine were removed from the testing apparatus, and 75% ethanol was used to eliminate odors.

### 2.3 Open field test

The open field test was used to assess the free exploratory behavior and anxiety levels of mice in a new environment (Walsh and Cummins, 1976). The experiment was conducted in a white plastic box (45 cm × 45 cm × 45 cm), with the center area (20 cm × 20 cm) designated as the central zone. We used 8 weeks-old male and female mice, with the following group sizes: CTR♂ *n* = 15, GDM♂ *n* = 15, CTR♀ *n* = 14, GDM♀ *n* = 11. During the test, mice were randomly placed into the box one by one to freely explore for 10 min. The total distance traveled by the mice was recorded to measure their locomotor activity, the time spent in the central zone was used to assess anxiety levels, and the grooming time throughout the test was used to evaluate the severity of stereotypic behavior in the mice. Two-way ANOVA was used for analyzing behavioral parameters, and Šídák’s multiple comparisons test was used as a *post-hoc* test to correct for multiple comparisons.

### 2.4 Elevated plus maze (EPM)

The elevated plus maze (EPM) was used to assess anxiety levels in mice (Danduga and Kola, 2024). The maze was elevated 1 m above the ground and consists of a central platform, two enclosed arms, and two open arms. We used 8 weeks-old male and female mice, with the following group sizes: CTR♂ *n* = 15, GDM♂ *n* = 15, CTR♀ *n* = 14, GDM♀ *n* = 11. Mice were individually placed on the central platform of the maze and allowed to explore freely for 5 min. The time each mouse spent in the open arms was recorded as a measure of anxiety levels. Two-way ANOVA was used for analyzing behavioral parameters, and Šídák’s multiple comparisons test was used as a *post-hoc* test to correct for multiple comparisons.

### 2.5 Marble burying test

The marble burying test was performed in standard transparent plastic cages filled with 5 cm of bedding. We used 9 weeks-old male and female mice, with the following group sizes: CTR♂ *n* = 8, GDM♂ *n* = 10, CTR♀ *n* = 9, GDM♀ *n* = 9. Each mouse was placed alone in a test cage containing 15 glass marbles for 30 min. After the 30 min test, the number of marbles buried by each mouse was recorded and converted into a percentage for statistical analysis. Two-way ANOVA was used for analyzing behavioral parameters, and Šídák’s multiple comparisons test was used as a *post-hoc* test to correct for multiple comparisons.

### 2.6 Novel object recognition

We used the Novel Object Recognition (NOR) test to evaluate the learning and memory abilities of mice. The experiment was conducted in a white plastic box (45 cm × 45 cm × 45 cm) and involved 9 weeks-old mice divided into two groups: CTR♂ (*n* = 15), GDM♂ (*n* = 15). The experiment consisted of three phases: Adaptation phase: Mice were allowed to freely explore the empty box for 10 min; Two identical objects (A) were placed in the box, and mice explored the objects for 10 minutes; One of the familiar objects was replaced with a novel object (B), and mice were allowed to explore for 5 min. The exploration time and discrimination index (DI) were used to assess the preference for the novel object. Differences between groups were analyzed to evaluate changes in cognitive function. Unpaired *t*-test statistical method was used to analyze the differences between two groups.


D⁢i⁢f⁢f⁢e⁢r⁢e⁢n⁢c⁢e⁢I⁢n⁢d⁢e⁢x=

$$\frac{(\mathrm{Time}\mathrm{spent}\mathrm{exploring}\mathrm{object}\left(B\right)-\mathrm{Time}\mathrm{spent}\mathrm{exploring}\mathrm{object}\left(A\right)}{(\mathrm{Time}\mathrm{spent}\mathrm{exploring}\mathrm{object}\left(\text{B}\right)+\mathrm{Time}\mathrm{spent}\mathrm{exploring}\mathrm{object}\left(A\right)}$$

### 2.7 Three-chamber social test

This test was used to assess the sociability and social novelty preference of mice by measuring their interaction time with different objects, reflecting their interest in social subjects (Jabarin et al., 2022). The experimental apparatus was a dark box divided into three connected areas (left, central, and right). Each of the left and right sides contained a cage for placing unfamiliar mice. We used 10 weeks-old male and female mice, with the following group sizes: CTR♂ *n* = 15, GDM♂ *n* = 15, CTR♀ *n* = 10, GDM♀ *n* = 9. The experiment consisted of three phases, each lasting 10 min. In the first phase, both cages were empty, and the test mouse was placed in the central area, allowing it to freely explore for 10 min. In the second phase, a stranger mouse (stranger 1) was placed in one of the cages, while the other remained empty. The test mouse was placed back in the central area and allowed to explore freely for another 10 min. In the third phase, a new stranger mouse (stranger 2) was placed in the previously empty cage, and the test mouse was again allowed to explore freely for 10 min. Measurement metrics were defined as follows:

1. Phase 2 Metrics:In the second phase, the contact time between the test mouse and Stranger 1 was recorded, as well as the contact time between the test mouse and the empty cage. These two contact times were compared to assess whether the test mouse showed a preference for Stranger 1 over the empty cage.
2. Phase 3 Metrics:In the third phase, the contact time between the test mouse and Stranger 2 was recorded, along with the contact time between the test mouse and Stranger 1. These contact times were compared to evaluate whether the test mouse showed a preference for the new stranger (Stranger 2) over the familiar one (Stranger 1).

In this step, a one-way ANOVA method was used to analyze male and female mice separately, and Tukey’s multiple comparisons test was used as a *post-hoc* test to correct for multiple comparisons.

For both phases, a difference index was calculated to quantify the preference of the test mouse (Leithead et al., 2024), the differential index analysis at each stage was performed using a two-way ANOVA to analyze:


D⁢i⁢f⁢f⁢e⁢r⁢e⁢n⁢c⁢e⁢I⁢n⁢d⁢e⁢x⁢(P⁢h⁢a⁢s⁢e⁢ 2)=

(Contact⁢Time⁢with⁢Stranger⁢ 1-Contact⁢Time⁢with⁢Empty⁢Cage)(Contact⁢Time⁢with⁢Stranger⁢ 1+Contact⁢Time⁢with⁢Empty⁢Cage)

D⁢i⁢f⁢f⁢e⁢r⁢e⁢n⁢c⁢e⁢I⁢n⁢d⁢e⁢x⁢(P⁢h⁢a⁢s⁢e⁢ 3)=

(Contact⁢Time⁢with⁢Stranger⁢ 2-Contact⁢Time⁢with⁢Stranger⁢ 1)(Contact⁢Time⁢with⁢Stranger⁢ 2+Contact⁢Time⁢with⁢Stranger⁢ 1)

### 2.8 Immunofluorescence staining

One hour after the completion of the three-chamber social test, mice were anesthetized with an intraperitoneal injection of 2% Avertin, followed by transcardial perfusion with 4% paraformaldehyde (PFA), and the entire mouse brain was fixed in 4% PFA for 24 h. Subsequently, the brain was washed with PBS and placed in a 30% sucrose solution for dehydration for 48 h. After complete dehydration, the whole brain was rapidly frozen using dry ice and sectioned into 50-micron thick slices using a Leica SM2010 R sliding microtome. The brain slices were stored in antifreeze solution at −20°C until use. In this experiment, we used 10 weeks-old male mice, with a sample size of: CTR♂ *n* = 3, GDM♂ *n* = 3.

Before performing immunofluorescence staining on the tissue sections, washed them twice with PBS and PBS-Tx (0.5% Triton X-100), each time for 5 min. Blocked the sections at room temperature with a blocking solution for 1 h. After blocking, incubated the tissue sections in a blocking solution containing the primary antibody overnight at 4°C. The next day, washed the sections in the sequence of PBS-Tx/PBS/PBS-Tx/PBS/PBS-Tx, for a total of five washes, each for 5 min. Then incubated them with a blocking solution containing the secondary antibody for 2 h at room temperature. Finally, rinsed once with PBS-Tx and three times with PBS, each for 10 min. Mounted the rinsed tissue sections onto glass slides and covered them using a mounting medium containing DAPI. Acquired images using the OLYMPUS VS120, and analyzed the fluorescence intensity using Fiji software (NIH, Bethesda, MD, United States). We analyzed the density of c-fos positive nuclei (c-fos-positive nuclei/mm^2^) and compared the two groups using the unpaired *t*-test statistical method.

### 2.9 Single nuclei isolation

The samples used in this experiment were the PFC of 10 weeks-old male mice, with a sample size of: CTR♂ *n* = 3, GDM♂ *n* = 3. Frozen tissue samples were homogenized using a glass dounce homogenizer in 3 mL of lysis buffer (10 mM Tris, pH 7.4, 10 mM NaCl, 3 mM MgCl_2_, and 0.05% (v/v) NP-40 detergent). The tissue was dounced to ensure thorough lysis. The sample volume was adjusted to a total of 5 mL with lysis buffer and incubated at room temperature for 5 min. To stop the lysis reaction, 5 mL of wash buffer (10 mM Tris, pH 7.4, 10 mM NaCl, 3 mM MgCl_2_, 1% BSA, 1 mM DTT, RNase inhibitor 1 U/μL, and nuclease-free water) was added. The sample was then filtered through a 30 μm cell strainer and centrifuged at 500 × g for 5 min to collect the nuclear pellet. The nuclei were resuspended in 10 mL of wash buffer, and this washing step was repeated three times. After washing, the nuclei were resuspended in 1 mL of wash buffer and mixed with 25% Optiprep. The mixture was carefully layered on top of a 29% Optiprep cushion and centrifuged at 10,000 × g for 30 min to isolate the nuclei. Finally, the nuclei were collected and washed three times to ensure the removal of any residual Optiprep.

### 2.10 Single cell sequencing and analysis

AO/PI (LUNA) counting and microscopic observation of nuclear status were performed. The nuclei were finally resuspended in Nuclei Resuspension Buffer, and the cell concentration was adjusted to a suitable concentration of 700–1,200 cells/μl. The cell suspension was loaded into Chromium microfluidic chips with 3′ v3.1 chemistry and barcoded with a 10X Chromium Controller (10X Genomics). The RNA from the barcoded cells was reverse-transcribed, and sequencing libraries were constructed using a Chromium Single Cell 3′ v3.1 reagent kit (10X Genomics) according to the manufacturer’s instructions. Sequencing was performed with Illumina (HiSeq 2000 or NovaSeq, depending on the project) platform according to the manufacturer’s instructions (Illumina).

Global analysis between samples: Based on the filtered gene expression matrix using Seurat, differential expression analysis between samples was carried out using the edgeR package (McCarthy et al., 2012) to obtain zone-specific marker genes. Enrichment analysis of marker genes: Gene Ontology (GO) enrichment analysis of marker genes was implemented using the clusterProfiler R package, in which gene length bias was corrected. GO terms with corrected *P*-values less than 0.05 were considered significantly enriched by marker genes. KEGG (Du et al., 2014) is a database resource for understanding high-level functions and utilities of the biological system^1^. We used the cluster Profiler R package to test the statistical enrichment of marker genes in KEGG pathways.

### 2.11 RNA isolation and quantitative real time-PCR (qPCR)

The samples used in this experiment were the PFC of 10 weeks-old male mice, with a sample size of: CTR♂ *n* = 4, GDM♂ *n* = 4. Total RNA was extracted from the PFC of mice using Trizol (TaKaRa, Otsu, Shiga, Japan, 9108). Reverse transcription was performed using the PrimeScript RT Reagent Kit (TaKaRa, RR037A). TB Green Premix Ex Taq and specific primers were then used. The relative expression levels were calculated using the 2^–ΔΔCq^ method, with β-Actin as the internal control. Comparison between CTR and GDM using the *t*-test analysis method. The primer sequences are listed in Supplementary Table 1.

### 2.12 UHPLC-MS

The samples used in this experiment were the PFC of 10 weeks-old male mice, with a sample size of: CTR♂ *n* = 3, GDM♂ *n* = 3.1 mL of precooled methanol/acetonitrile/water (v/v, 2:2:1) was added to the sample, sonicated in an ice bath for 1 h, then incubated at −20°C for 1 h. Centrifuged at 16,000 g, 4°C for 20 min, and the supernatant was transferred to a sampling vial for LC-MS analysis. Additionally, quality control (QC) samples were prepared by mixing equal amounts of all samples to ensure the quality of metabolic profiling and data normalization. The dried extracts were dissolved in 50% acetonitrile, filtered through a disposable 0.22 μm cellulose acetate filter, transferred into 2 mL HPLC vials, and stored at −80°C until analysis.

We performed chromatographic and mass spectrometric detection on the LC/MS platform (Cao et al., 2020). This platform is based on a Shimadzu Nexera X2 LC-30AD system equipped with an Acquity UPLC HSS T3 column (1.8 μm, 2.1 × 50 mm, Waters) and a triple quadrupole mass spectrometer (5500 QTRAP, AB SCIEX). Metabolites were detected using electrospray ionization in both negative-ionization and positive-ionization modes. In chromatographic detection, the column temperature was maintained at 40°C, with a flow rate of 200 μL/min. During sample acquisition, 5 μL samples were sequentially injected using the LC autosampler, and a QC sample was injected every few samples. The compound separation method was gradient elution: the gradient consisted of 0.1% formic acid aqueous solution (solvent A) and 100% acetonitrile (solvent B). It started with 100% solvent A for 2.5 min, then increased linearly to 70% solvent A over 9 min, followed by a linear increase to 0% solvent A over 1 min, with a 5.4 min hold, before returning to the initial mixture within 0.1 min and re-equilibrating for 2.5 min.

The MS conditions were set as follows: negative-ionization: Source Temperature 550°C, Ion Source Gas1 (GAS1): 40, Ion Source Gas2 (GAS2): 50, Curtain Gas (CUR): 35, Ion Spray Voltage Floating (ISVF): −4500V;positive-ionization: Source Temperature 550°C Ion Source Gas1 (GAS1): 40, Ion Source Gas2 (GAS2): 50, Curtain Gas (CUR): 35, Ion Spray Voltage Floating (ISVF): 5500V. Transition were detected by MRM mode.

The discriminating metabolites were obtained using a statistically significant threshold of variable influence on projection (VIP) values obtained from the OPLS-DA model and two-tailed Student’s *t*-test (*p*-value) on the normalized raw data. Metabolites with VIP greater than 1 and *p*-value less than 0.05 were considered to be statistically significant metabolites. The identified differential metabolites were used to perform cluster analyses with R package. To identify the perturbed biological pathways, the differential metabolite data were performed KEGG pathway analysis using KEGG database^2^. KEGG enrichment analyses were carried out with the Fisher’s exact test, and FDR correction for multiple testing was performed. Enriched KEGG pathways were nominally statistically significant at the *p* < 0.05 level.

### 2.13 DNA methylation

Genomic DNA was extracted from the PFC of mice using the TIANmap Micro DNA kit (Tiangen, Beijing, China). The samples used in this experiment were the PFC of 10 weeks-old male mice, with a sample size of: CTR♂ *n* = 3, GDM♂ *n* = 3. Bisulfite conversion was performed strictly according to the manufacturer’s instructions using the EpiTect Bisulfite kit (Qiagen). The specificity of the bisulfite DNA PCR products was checked by agarose gel analysis. Pyrosequencing was conducted using a PyroMark Q24 pyrosequencer (Qiagen) with the PyroMark Gold Q24 reagent kit (Qiagen) and 10 pmol of sequencing primer. The experimental data were analyzed using PyroMark Q24 software (Qiagen). The pyrosequencing primers were designed using the Qiagen PyroMark Assay Design 2.0 software (Qiagen).

### 2.14 Statistical analysis

For each figure, a statistical test matching the structure of the experiment and the structure of the data was used. Variances within each group of data are shown as standard error of the mean (SEM). *P < 0.05, **P < 0.01, ***P < 0.001. Statistical analysis was performed using GraphPad Prism software (version 9.0.0, GraphPad Software Inc., San Diego, CA, United States).

## 3 Results

### 3.1 GDM induces repetitive behavior in male offspring

To evaluate the impact of GDM on the overall health and behavioral changes of offspring, we conducted the following experiments. As body weight is an important indicator of overall health and metabolic status, we first monitored the body weight of offspring after weaning. Body weight monitoring showed that there were no significant differences in the body weight of male and female mice between the GDM group and the CTR group from 3 to 8 weeks (Figure 1C). To investigate the effects of GDM on offspring anxiety and motor ability, offspring were subjected to Open Field (OF) and Elevated Plus Maze (EPM) tests in 8 weeks. The experimental results showed that, compared to the CTR group, both male and female offspring of GDM exhibited no significant differences in total distance in the OF test, as well as no significant differences in the time spent in the center of the arena or in the open arms of the EPM. This experiment suggests that, regardless of sex, offspring of GDM showed no differences in motor ability or anxiety levels compared to the control group (Figures 1D, F). Grooming behavior is a widely used metric to evaluate stereotyped and repetitive actions in rodents (Silverman et al., 2010). To specifically examine repetitive behaviors, which are relevant to ASD, we recorded the grooming time for each mouse in the open field. The results showed a significant increase in grooming time in male offspring of the GDM group, while no significant change was observed in females (Figure 1E). To further validate the occurrence of repetitive behaviors, we conducted a marble-burying test, another behavioral test for stereotyped behaviors. The results indicated that male mice in the GDM group showed a significantly higher percentage of marbles buried compared to the CTR group (Figures 1G–I). These results collectively indicate that GDM induced an increase in repetitive behaviors in male offspring.

### 3.2 GDM induces social defects in offspring

**FIGURE 2:**
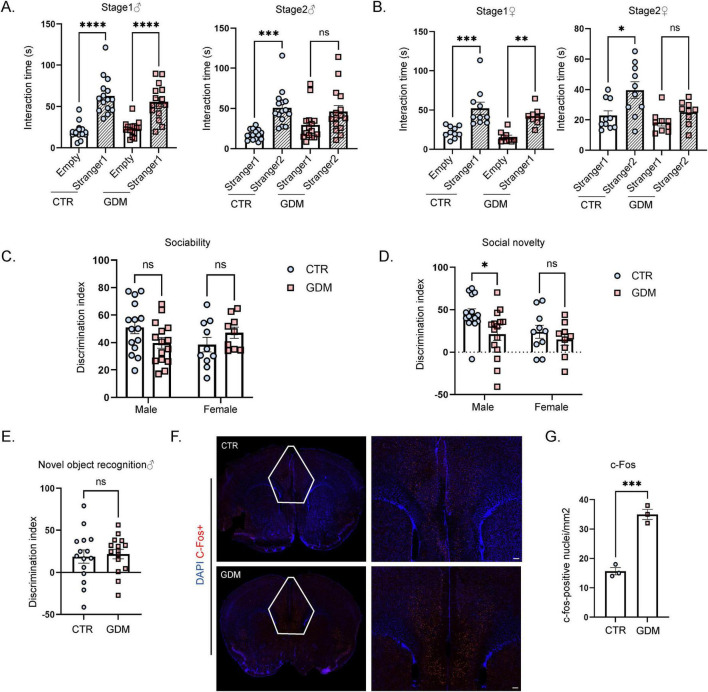
Gestational diabetes mellitus (GDM) male offspring exhibited impaired social novelty and abnormal neuronal activation in the prefrontal cortex. **(A)** This figure shows the differences in social interaction time between the two sides for male mice (*n* = 15/group) during the first and second phases of the three-chamber social test. Comparisons between experimental groups were analyzed by one-way ANOVA, ***p < 0.001, ****p < 0.0001. Stage 1: F (3, 56) = 26.12, *p* < 0.0001; Stage 2: F (3, 56) = 7.920, *p* = 0.0002. **(B)** This figure shows the differences in social interaction time between the two sides for female mice during the first and second phases of the three-chamber social test (CTR: *n* = 10; GDM: *n* = 9). Comparisons between experimental groups were analyzed by one-way ANOVA, *p < 0.05, **p < 0.01, ***p < 0.001. Stage 1: F (3, 34) = 12.95, *p* < 0.0001; Stage 2: F (3, 34) = 6.485, *p* = 0.0014. **(C)** The social discrimination index of male mice (*n* = 15/group) and female mice (CTR: *n* = 10; GDM: *n* = 9) during the first phase. **(D)** Social discrimination index of male mice (*n* = 15/group) and female mice (CTR: *n* = 10; GDM: *n* = 9) during the second phase. In panels **(C,D)**, data were analyzed by two-way ANOVA, F_interaction_ (1, 45) = 1.150, *p* = 0.2892; F_sex_ (1, 45) = 3.829, *p* = 0.0566; F_GDM_ (1, 45) = 5.457, *p* = 0.0240, followed by Šídák’s multiple comparisons to analyze the effects produced by GDM, *p < 0.05, t = 2.739, df = 45.00, *p* = 0.0175. **(E)** This figure shows the discrimination index of novel object recognition of male offspring (*n* = 15/group), comparisons between experimental groups were analyzed by unpaired *t*-test. **(F)** This figure displays the signal intensity of c-fos in neurons within the prefrontal cortex following immunofluorescence staining (red for c-fos, blue for DAPI), scale bar, 50 μm. **(G)** The density of c-fos-positive nuclei in the prefrontal cortex (PFC) of male mice (*n* = 3/group). Comparisons between experimental groups were analyzed by unpaired *t*-test, ***p < 0.001, t = 9.171, df = 4, *p* = 0.0008. Data are presented as mean ± SEM.

To investigate the impact of GDM on the social abilities of offspring, we conducted three-chamber social interaction experiment. In the first phase of the sociability test, both the GDM group and the CTR group mice spent more time interacting with the stranger mouse than with the empty cage. This result indicates that mice in the GDM group exhibited normal sociability. In the second phase of the test, which measures social novelty by evaluating the preference between a familiar and an unfamiliar mouse, male and female mice in the GDM group spent a similar amount of time with both the unfamiliar and the familiar mouse (Figures 2A, B). However, when analyzing the discrimination index, only male mice in GDM group showed a significant difference compared to CTR group (Figures 2C, D). These findings suggest that compared to CTR group, the social novelty of the GDM group is impaired, with a more pronounced effect observed in male mice. To rule out the potential influence of cognitive impairments on the results of the three-chamber social test, we conducted a novel object recognition (NOR) test, which assesses spatial memory and cognitive abilities. The results showed no significant differences in spatial memory performance between male offspring in the GDM and CTR groups, suggesting that the observed social deficits are not secondary to impairments in cognitive abilities (Figure 2E). To further explore the neural basis underlying the observed social behavior impairments, we examined neural activity in the PFC, a key brain region involved in social behaviors (Mediane et al., 2024). Immunofluorescence staining for c-fos, a marker of neuronal activation, was performed on brain sections collected one hour after the three-chamber social interaction experiment. The results revealed a significant increase in the density of c-fos-positive nuclei in the PFC of male mice in the GDM group after social behavior stimulation (Figures 2F, G). This abnormal activation in the PFC may underlie the social novelty deficits observed in male GDM offspring.

These findings demonstrate significant impairments in social novelty recognition observed in males, and this is associated with abnormal activation of the PFC after social interaction.

### 3.3 Single-cell transcriptome analysis of the offspring prefrontal cortex

The prefrontal cortex (PFC), as demonstrated by numerous studies, is a brain region closely associated with emotions and social behavior (Mediane et al., 2024). Disruptions in the PFC, particularly an imbalance between excitatory and inhibitory neuronal activity, have been linked to social behavioral disorders and autistic-like behaviors (Yizhar et al., 2011). These findings suggest that alterations in the PFC may underlie behavioral changes observed in offspring.

**FIGURE 3:**
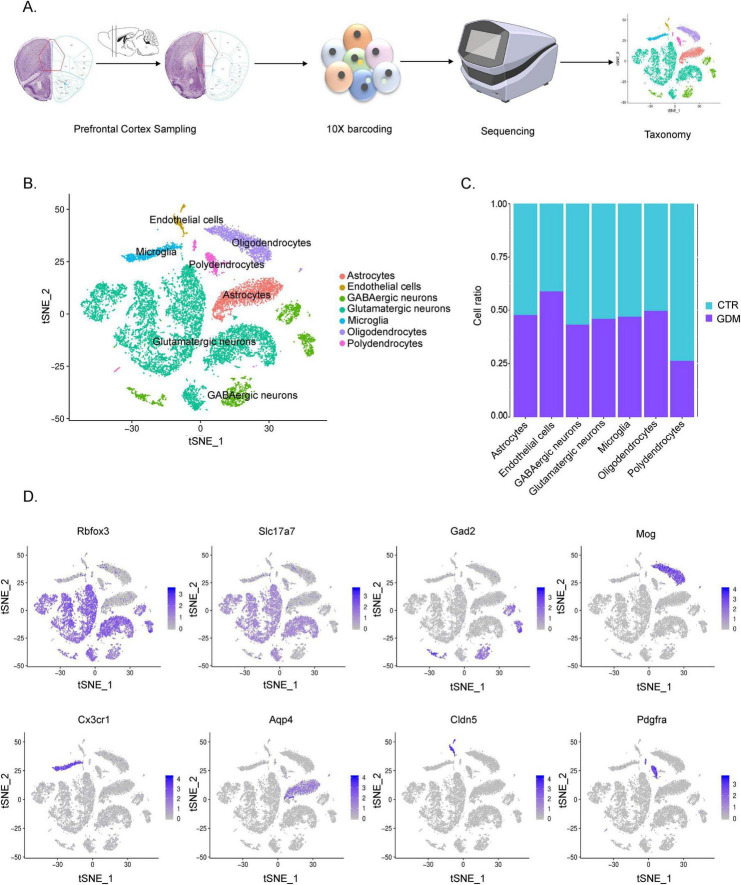
Single-cell transcriptome analysis of the gestational diabetes mellitus (GDM) offspring prefrontal cortex. **(A)** Single-cell RNA sequencing workflow diagram. **(B)** t-SNE of cells measured by snRNA-seq, cells are colored by cell-type assignment. **(C)** Proportion of each cell type in GDM and CTR. **(D)** Expression of cell type-specific markers in each broad cell cluster color-highlighted on t-SNE plots.

Given the role of the PFC in regulating social behaviors, we hypothesize that GDM-induced changes in the PFC may be associated with behavioral alterations in offspring. To investigate this, we performed single-cell transcriptome sequencing on PFC tissue from male offspring to explore potential molecular and cellular mechanisms that could link GDM to these behavioral changes. The results of analysis showed that a total of 2,0114 cells (10844 from CTR samples and 9,270 from GDM samples) were obtained to perform scRNA-seq. A total of 24 cell clusters were identified in accordance with their representative cell markers (Figure 3A). By reviewing the literature, we manually annotated these cells into seven categories. Firstly, we use Rbfox3 as a marker to classify the cells into neurons and non-neuronal cells. Secondly, based on the expression of cell type-specific markers, the non-neuronal cells were clustered as: oligodendrocyte (Mog+), astrocytes (Aqp4+), polydendrocytes (Pdgfra+), microglia (Cx3cr1+), endothelial cells (Cldn5+). Clusters 0,1,3,4,6,7,11 and 12 were designated as the glutamatergic neurons with positive Gad2 expression (Figures 3B, D). Analysis of the proportions of each cell group revealed that, the GDM group showed an increased proportion of epithelial cells, a decreased proportion of oligodendrocyte precursor cells, and slight decreases in the proportions of and GABAergic neurons (Figure 3C). These findings suggest that GDM alters the cellular composition of the PFC, particularly affecting oligodendrocyte precursor cells and GABAergic neurons, which may contribute to the imbalance in excitatory and inhibitory neuronal activity observed in GDM offspring.

These results indicate that GDM induces significant changes in the cellular composition of the PFC, including alterations in inhibitory neurons and glial cell populations, which may underlie the disrupted neural circuits and social behavioral deficits observed in GDM offspring.

### 3.4 Alterations in GABAergic neuron subpopulations in the PFC of GDM offspring

**FIGURE 4:**
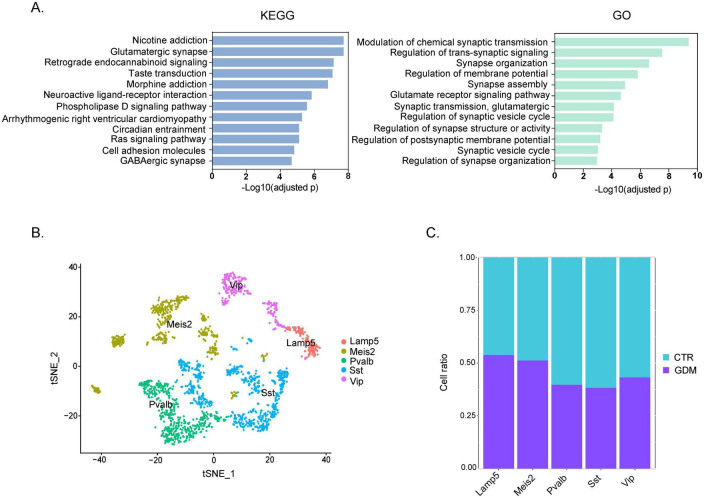
Pathway enrichment and GABAergic neuronal subclusters. **(A)** Bar plots of KEGG enrichment analysis and Gene Ontology (GO) Biological Process. The x-axis represents the -Log10 (adjusted p) of significantly enriched pathways, and the y-axis shows the names of the enriched pathways. Each bar is sorted by enrichment score from high to low. **(B)** t-SNE plot showing that GABAergic neurons of PFC can be broadly classified into five unique subtypes. **(C)** Proportion of each GABAergic neuron subtype in GDM and CTR.

To further investigate the biological mechanisms involved in the abnormal behavior of GDM-F1 offspring, we performed KEGG and GO analyses on the differentially expressed genes obtained from scRNA sequencing. Abnormal behaviors, particularly those related to hyperactivity or social impairments, are often linked to imbalances in neuronal excitation and inhibition (Sohal and Rubenstein, 2019), prompting us to investigate synapse-related pathways and neuronal subpopulations. The KEGG enrichment analysis indicates that the altered pathways are enriched in “glutamatergic synapse” and “GABAergic synapse” (Figure 4A). The GO enrichment analysis shows that the pathways are mainly concentrated in synapse-related pathways (Figure 4A). The results of The results of KEGG and GO enrichment analyses suggest potential abnormalities in the excitatory-inhibitory system in the prefrontal cortex (PFC). Therefore, we further performed subpopulation analysis on GABAergic neurons. We further divided the inhibitory neurons into five subgroups (Figure 4B): Lamp5 interneurons (IN-Lamp5), Meis2 interneurons (IN-Meis2), parvalbumin interneurons (IN-PV), somatostatin interneurons (IN-SST), and VIP interneurons (IN-VIP). Analyzing the cell proportions of each subgroup of inhibitory neurons revealed that in the inhibitory neurons, the number of cells in the PV-IN and SST-IN subgroups decreased in the GDM group compared to the control group, with a more pronounced decrease in the SST-IN subgroup (Figure 4C). Given the critical role of SST interneurons in modulating pyramidal neuron activity and regulating cortical circuit excitability, this reduction may contribute to the hyperexcitability observed in the PFC of GDM offspring.

These findings suggest that the abnormal behaviors observed in GDM offspring may stem from synaptic dysfunction and a disruption in the excitatory-inhibitory balance within the PFC, particularly driven by reductions in SST and PV interneurons.

### 3.5 Metabolites alterations in the prefrontal cortex of GDM offspring

**FIGURE 5:**
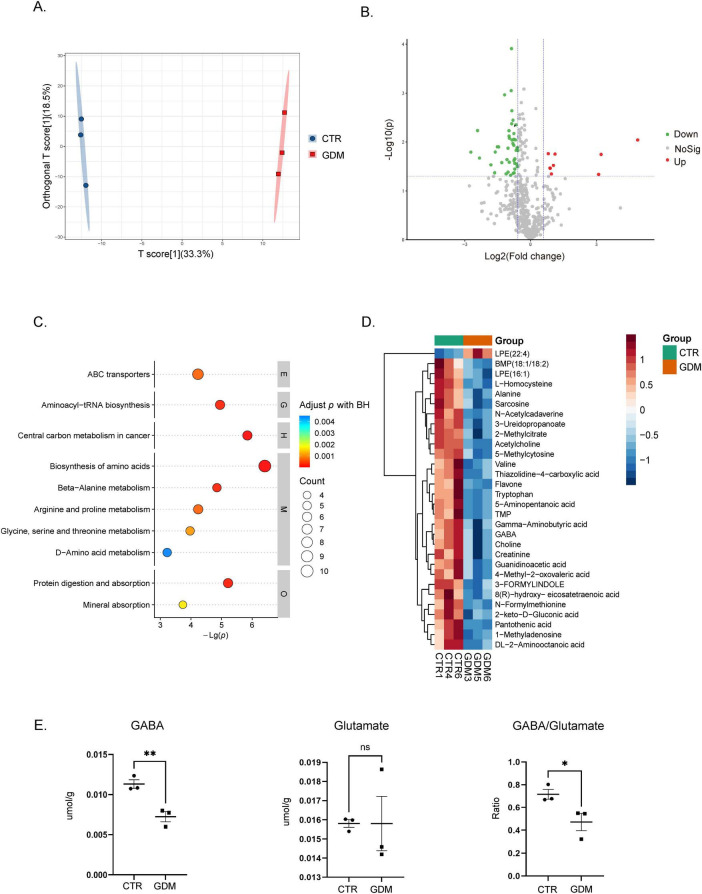
Metabolic profiling of the mouse prefrontal cortex. **(A)** The figure shows the metabolic profile differences between the gestational diabetes mellitus (GDM) group and the CTR group using orthogonal partial least squares discriminant analysis (OPLS-DA). **(B)** The volcano plot displays the distribution of metabolite differences between the GDM group and the CTR group. Each point in the figure represents a metabolite, plotted according to log2 Fold Change and adjusted *p*-value. The significance threshold is set at *p* < 0.05, the vertical dashed lines indicate log2 (1/1.5) and log2 (1.5). **(C)** This figure shows the results of KEGG pathway enrichment analysis based on metabolite data. The vertical axis represents pathway names, while the horizontal axis represents -Log(p). **(D)** The heatmap shows the relative abundance changes of the top 30 significantly different metabolites across the different experimental groups. **(E)** The content of GABA and glutamate, as well as the ratio of GABA to glutamate, in the PFC of the GDM group and the CTR group. Comparisons between experimental groups were analyzed by unpaired *t*-test, *p < 0.05, ***p < 0.01. GABA content: t = 4.970, df = 4, *p* = 0.0077. The ratio of GABA to glutamate: t = 2.836, df = 4, *p* = 0.0471. Data are presented as mean ± SEM.

Alterations in brain metabolites is often associated with neurodevelopmental disorders and may contribute to behavioral and neural abnormalities in offspring (Notarangelo and Pocivavsek, 2017). To investigate whether GDM influences the metabolic profile of offspring, we performed metabolomic profiling to analyze the metabolic changes in the PFC. As shown in the metabolic data plots, a clear shift in the metabolic profile was observed in the GDM offspring compared to CTR (Figure 5A), indicating significant alterations in metabolite composition. As shown, the metabolic data plots displayed a distinguishable shift of the metabolic profile in in GDM offspring (Figure 5A). Next, we used the criteria of VIP > 1 in the OPLS-DA model and *P*-value < 0.05 to screen for significantly different metabolites, followed by an analysis of their expression changes and functional pathway analysis. In our data, a total of 16 significantly upregulated metabolites and 79 significantly downregulated metabolites were identified in the GDM group (Figure 5B). To further understand the biological implications of these changes, we conducted KEGG pathway enrichment analysis. The analysis showed that the top 10 enriched pathways included amino acid synthesis, alanine, aspartate, and glutamate metabolism, arginine and proline metabolism, and beta-alanine metabolism, among others (Figure 5C). These pathways are critical for maintaining normal neuronal function and neurotransmitter balance.

Notably, two of the enriched pathways were directly related to GABA, an important inhibitory neurotransmitter. Heatmap analysis revealed that GABA levels were significantly downregulated in the GDM group (Figure 5D). To confirm these findings, we quantified the concentrations of GABA and glutamate in the PFC of 8 weeks-old GDM offspring. The results showed that GABA concentration was significantly reduced in the PFC of the GDM group, whereas glutamate concentration remained unchanged, leading to a decreased GABA-to-glutamate ratio (Figure 5E).

These results highlight a significant alteration in the metabolic profile, particularly involving the glutamate/GABAergic system, which may play a critical role in the neurobehavioral deficits associated with GDM.

### 3.6 GDM induced hypermethylation and decreased expression of GAD2

**FIGURE 6:**
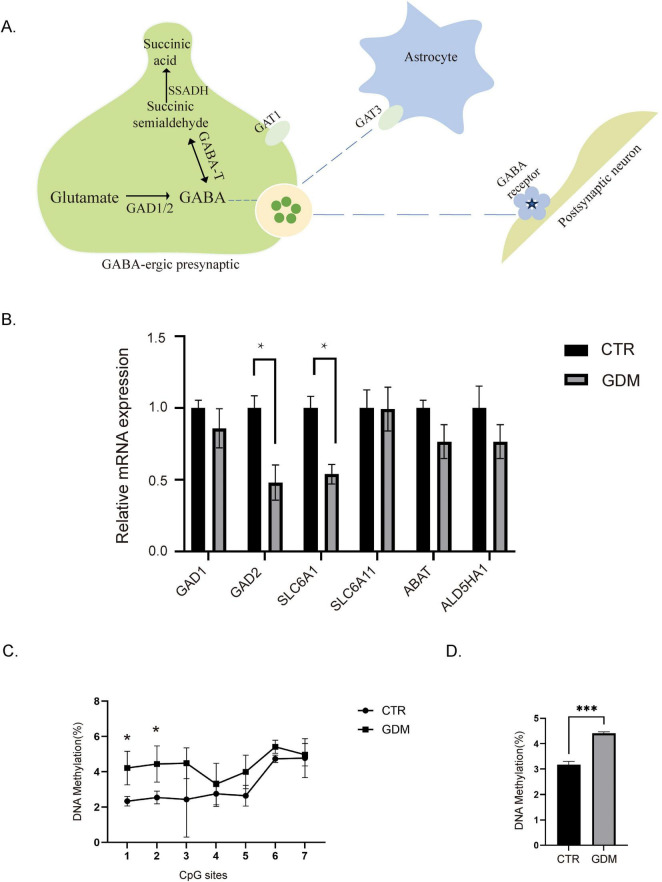
Expression levels and methylation status of GAD2. **(A)** Schematic diagram of GABA metabolism and transport processes. **(B)** The mRNA levels for gene expression of key enzymes in the GABA synthesis pathway (*n* = 4/group). Comparisons between experimental groups were analyzed by unpaired *t*-test. *p < 0.05. GAD2: t = 3.473, df = 6, *p* = 0.0133. SLC6A1: t = 4.318, df = 4.*p* = 0.0125. **(C)** Methylation status of GAD2 promoter regions, in each CpG site in the prefrontal cortex (PFC) of male offspring (*n* = 3/group). Comparisons between experimental groups were analyzed by two-way ANOVA, F (1, 28) = 21.40, *P* < 0.0001, followed by Šídák’s multiple comparisons, *p < 0.05, t1 = 2.90, df = 28.00, *p* = 0.0483; t2 = 2.939, df = 28.00, *p* = 0.0448. **(D)** Overall methylation levels of the GAD2 promoter region in the PFC of male offspring (*n* = 3/group). Comparisons between experimental groups were analyzed by unpaired *t*-test. ***p < 0.001. t = 8.950, df = 4, *p* = 0.0009. Data are presented as mean ± SEM.

The neurotransmitter GABA plays a critical role in maintaining the balance of excitatory and inhibitory signaling in the brain, and disruptions in its synthesis or regulation are associated with various behavioral abnormalities, including those observed in ASD (Zhao et al., 2021). Given that GABA concentration was significantly decreased in the PFC of GDM offspring, we sought to investigate the molecular mechanisms underlying this reduction. To address this, we conducted qPCR analysis to examine the expression of key enzymes involved in GABA synthesis and transport pathways. GABA is synthesized from glutamate through decarboxylation by GAD1 and GAD2. It is further metabolized by GABA -T and SSADH into succinate, an intermediate in the tricarboxylic acid cycle. Additionally, GABA in the synaptic cleft can be reabsorbed into neurons or glial cells via GABA transporters (GAT) (Figure 6A). Through qPCR analysis, we found that both GAD2 and GAT1 (SLC6A1) mRNA levels were significantly reduced in the PFC of 8 weeks-old offspring from the GDM group compared to the control group. Meanwhile, no significant differences were observed for other enzymes (Figure 6B). This suggests that the decrease in GABA concentration is caused by the reduced expression levels of key enzymes involved in the GABA synthesis pathway.

Studies have shown that the GAD2 gene is regulated by methylation (Tang et al., 2023). In individuals with ASD, changes in the methylation levels of GAD2 may affect its expression, thereby influencing neurodevelopment and behavior. Therefore, we designed primers targeting the CpG island in the promoter region of GAD2 and measured its methylation levels. We performed pyrosequencing to analyze the methylation levels of the GAD2 promoter region. The results showed that the overall methylation levels of the GAD2 promoter region increased in the GDM group (Figure 6D), with the most significant changes observed in the 1st and 2nd CpG islands (Figure 6C).

Our results suggest that the reduced GABA concentration in the PFC of GDM offspring is driven by decreased expression of GAD2 and GAT1, likely mediated by epigenetic modifications, particularly hypermethylation of the GAD2 promoter. These molecular changes may play a crucial role in the disrupted GABAergic system and behavioral deficits observed in GDM offspring.

## 4 Discussion

The DOHaD hypothesis suggests that environmental factors during early development, such as prenatal nutrition and maternal health, can affect the development of the fetus, influencing the individual’s health in adulthood (Gluckman et al., 2010). The development of the nervous system during prenatal and early postnatal periods is susceptible to environmental factors (Doi et al., 2022). Several studies have demonstrated that adverse early-life environmental factors, such as maternal immune activation and high-fat diets during pregnancy, can profoundly impact fetal neurodevelopment and behavior (Ceasrine et al., 2022; Hayes et al., 2022). Epidemiological evidence has shown that GDM, as a prevalent metabolic disorder during pregnancy, can induce abnormal neurodevelopment in offspring and increase the risk of various neurological disorders including ASD (Liu et al., 2024). In our study, we established a gestational intrauterine hyperglycemia model at 10.5 days of pregnancy to simulate the effects of mid-to-late pregnancy intrauterine hyperglycemic exposure on offspring. We found that male offspring of GDM exhibited significantly increased stereotypic behavior in the open field test and marble burying test, and significantly decreased social novelty in the three-chamber social test. This can be summarized as male offspring of gestational diabetes mothers displaying autistic-like behaviors. In our experiment, we did not observe significant phenotypic changes in the female offspring of the GDM group, as were observed in the male offspring. We speculate that this result of sexual dimorphism may be related to multiple factors. Previous studies have demonstrated that male fetuses exposed to prenatal and perinatal adversities tend to show more severe developmental impairments during early childhood compared to female fetuses (DiPietro and Voegtline, 2017). Researchers suggest that this may be attributed to sex hormones, intrinsic structural differences, placental factors, and epigenetic modifications. Additionally, the incidence of autism itself is higher in males than in females (Ferri et al., 2018). Some researchers believe that testosterone may interact with downstream molecules such as neurotransmitters, neuropeptides, and immune pathways, thereby contributing to male vulnerability (Luo et al., 2023). Finally, regarding how GDM leads to sexually dimorphic behavioral outcomes, we believe that the underlying mechanisms require further experimental investigation.

Neurodevelopmental outcomes are strongly influenced by the interplay between the developing fetal brain and the maternal metabolic environment. In particular, the PFC, as a critical brain region associated with social behavior, is highly sensitive to disruptions during development (Bellard et al., 2023). It plays a pivotal role in integrating information necessary for complex social interactions, decision-making, and emotional regulation (Bellard et al., 2023). Research indicates that changes in the development or function of the PFC can result in social deficits and other autistic-like behaviors, emphasizing its susceptibility to environmental factors during critical developmental periods (Kolb et al., 2012). Numerous studies have shown that GDM can affect offspring brain development through various pathways, such as influencing the fetal brain fatty acid profile via the placenta, impacting microglial activation through oxidative stress, and altering neural circuits in the brain (Araujo et al., 2015; Vuong et al., 2017; Bataveljic et al., 2024; Cantacorps et al., 2024). Neuronal network hyperexcitability is associated with impairments in social behavior and various neurodevelopmental issues (Antoine et al., 2019; Bataveljic et al., 2024). The normal function of the brain requires a balance between excitatory (E) and inhibitory (I) neurotransmitters. E/I imbalance are considered a key factor contributing to social behavior deficits and Stereotypic behavior (Yizhar et al., 2011; Harris et al., 2016; Sohal and Rubenstein, 2019). In our study, we found excessive neuronal activation in the PFC of male mice in the GDM group after social interaction. Using single-cell RNA sequencing, we observed a reduction in the number of somatostatin (SST) inhibitory interneurons among GABAergic neurons in the GDM group. GABAergic neurons play a crucial role in regulating excitatory signal transmission by inhibiting nearby glutamatergic pyramidal neurons, which helps control the generation and timing of action potentials (Chen et al., 2019). Dysregulation of GABAergic neurons can lead to increased neuronal activity and excitability, resulting in social behavior impairments.

Further, we conducted a metabolomic analysis of the PFC in 8 weeks-old male offspring, which revealed a significant reduction in GABA concentration and a decrease in the GABA/glutamate ratio in the PFC. This confirms the disruption of the GABAergic system in male offspring. To further investigate the molecular mechanisms underlying this disruption, we conducted qPCR analysis on key enzymes involved in the synthesis, metabolism, and transport of GABA. The results showed a decrease in the mRNA levels of GAD2 and GAT1. GAD2, also known as GAD65, is one of the two isoforms of glutamate decarboxylase (Lee et al., 2019). It is specifically present in inhibitory neurons and catalyzes the decarboxylation of glutamate to produce GABA, thereby regulating neuronal excitability. Dysfunction of the GAD2 gene may lead to insufficient production of GABA, resulting in hyperactivity of the nervous system. This hyperactivity can trigger various neurological disorders, including autism (Kakizaki et al., 2021). Research has indicated that the expression level of GAD2 can be regulated by DNA methylation in its promoter region (Labouesse et al., 2015). To further investigate the reasons behind the reduced levels of GAD2 in the PFC of offspring from the GDM group, we conducted pyrosequencing to analyze the methylation levels of the GAD2 promoter region. Our findings showed an increase in methylation levels within this region. This suggests that GDM can regulate the expression of the GAD2 gene by affecting the methylation status of its promoter region in the PFC of male offspring, which may ultimately lead to dysfunction of the GABAergic system in these male offspring.

Our findings highlight the GABAergic system’s vulnerability to GDM and its crucial role in shaping neurodevelopmental trajectories. This study demonstrates disruptions in GABA concentration, neuronal excitability, and epigenetic regulation, providing new insights into how maternal metabolic disorders affect the development of the offspring’s brain. These disruptions in the PFC may be a key mechanism underlying the emergence of autistic-like behaviors in male offspring. Additionally, our results align with the increasing evidence that emphasizes the importance of the maternal metabolic environment in determining long-term neurodevelopmental outcomes.

In addition to advancing our understanding of the mechanisms underlying GDM-induced neurodevelopmental abnormalities, these findings have potential clinical implications. Addressing maternal metabolic health and exploring therapies that restore GABAergic function in the fetal or early postnatal brain may help alleviate the negative impacts of GDM on offspring neurodevelopment. Such interventions may provide promising opportunities for the early screening and therapeutic strategies for neurodevelopmental disorders, including ASD.

## 5 Conclusion

In this study, we observed impaired social abilities and stereotyped behaviors, two autistic-like behaviors, in male offspring. We further revealed that hyperglycemia in the mid-to-late pregnancy can disrupt the GABAergic system in the PFC of male offspring, including the synthesis of GABA neurotransmitters and the number of GABA neurons, leading to increased excitability in the PFC of male offspring. Therefore, early prevention, diagnosis, and treatment of gestational diabetes may be an important approach to reducing the risk of autism in offspring.

## Data Availability

The Single-cell transcriptome sequencing data are accessible in the BioProject PRJNA1216729 of the Sequence Read Archive (SRA) database (https://www.ncbi.nlm.nih.gov/sra/PRJNA1216729).

## Footnotes

1
http://www.genome.jp/kegg/

2
http://kegg.jp
