# Supplementary material for: Gestational diabetes induces autistic-like behaviors in offspring by disrupting the GABAergic system
Source: Front Neurosci. 2025 Feb 12;19:1538115. doi: 10.3389/fnins.2025.1538115 (PMC11861160; doi:10.3389/fnins.2025.1538115)
Supplement: Supplementary file 1 [file Table_1.docx]

**Supplementary Table 1. Primers are used for RT-qPCR.**

| **Primer**  **Symbol** | **Gene name** | **Primer direction** | **Sequences (5’to 3’)** | **Accession** |
| --- | --- | --- | --- | --- |
| *Gad1* | Glutamate decarboxylase 1 | Forward | AAGAACGGGGAGGAGCAAACTG | NM_001312900.2 |
|  |  | Reverse | ACTGGTGTGGGTGGTGGAAATC |  |
| *Gad2* | Glutamic acid decarboxylase 2 | Forward | CTGGTTAGAGAGGAGGGACTGATG | NM_008078.2 |
|  |  | Reverse | GTCATAGGACAGGTCATAGTGCTTATC |  |
| *Slc6a1* | Solute carrier family 6 | Forward | CTGTGCTGTCCTTCTGGCTGAAC | NM_178703.5 |
|  |  | Reverse | TTCCACGGGTTGTCACACTGTTTC |  |
| *Slc6a11* | Solute carrier family 6 | Forward | GGTGGAGTTCGTGTTGAGCGTAG | NM_172890.3 |
|  |  | Reverse | ACACCACGTAAGGAATCAGGAATGC |  |
| *Abat* | 4-aminobutyrate aminotransferase | Forward | CTGTGCTGTCCTTCTGGCTGAAC | NM_001170978.1 |
|  |  | Reverse | TTCCACGGGTTGTCACACTGTTTC |  |
| *Ald5ha1* | Aldhehyde dehydrogenase family 5 | Forward | CGGTTGTGACGGGCGGAAAG | NM_007393.5 |
|  |  | Reverse | TGCCAGAGGTCCAAAGGTCTCC |  |
| *β-Actin* | Actin, beta | Forward  Reverse | CATTGCTGACAGGATGCAGAAGG  TGCTGGAAGGTGGACAGTGAGG | NM_001289726.1 |
